# From currents to water masses: fine-scale insights into microbial biogeography in the Kuroshio–Oyashio Extension region

**DOI:** 10.1128/aem.01960-25

**Published:** 2025-12-11

**Authors:** Rong Huang, Yulin Zhang, Lulu Han, Ronghua Liu, Xinyi Zhai, Ke Zeng, Guodong Song, Honghai Zhang, Peng Yao, Zhaohui Chen, Jiwen Liu, Xiao-Hua Zhang

**Affiliations:** 1 Frontiers Science Center for Deep Ocean Multispheres and Earth System, and College of Marine Life Sciences, Ocean University of China 12591, Qingdao, China; 2 Laboratory for Marine Ecology and Environmental Science, Qingdao Marine Science and Technology Center 554912 , Qingdao, China; 3 Key Laboratory of Marine Chemistry Theory and Technology (Ministry of Education), Ocean University of China 12591, Qingdao, China; 4 Key Laboratory of Physical Oceanography, Ministry of Education, Ocean University of China 12591, Qingdao, China; 5 Key Laboratory of Evolution & Marine Biodiversity (Ministry of Education) and Institute of Evolution & Marine Biodiversity, Ocean University of China 12591, Qingdao, China; University of Delaware, Lewes, Delaware, USA

**Keywords:** microbial community, Kuroshio–Oyashio Extension (KOE) region, water mass

## Abstract

The Kuroshio–Oyashio Extension (KOE) region is a highly variable region in the North Pacific Ocean, characterized by strong environmental gradients and multi-scale oceanographic processes. However, the fine-scale impact of these currents and associated water masses on microbial communities remains poorly understood. Here, high-resolution samples from 18 to 24 layers were collected along a transect in the KOE region in 2021, with 16S rRNA gene amplicon sequencing and environmental parameter measurements conducted to investigate the Kuroshio–Oyashio influence on microbial communities. Strong regional and vertical variations in environmental parameters and microbial communities were observed, with main horizontal regional differentiations confined to the upper 500 m. Photoautotrophic and oligotrophic taxa (e.g., SAR11 clade and *Cyanobacteria*) were enriched in warm, oligotrophic Kuroshio region, whereas the cold nutrient-rich Oyashio and confluence regions supported higher microbial abundance, diversity, and complex microbial interactions. Consistently, heterotrophic bacteria (1.00 × 10⁶–1.17 × 10⁹ cells L⁻¹) were more abundant in the upper 55 m of the Oyashio and confluence regions than in the Kuroshio region. Below the thermocline (~500 m), community composition was primarily structured by depth, indicating a diminishing Kuroshio–Oyashio current influence. Three main water masses (subtropical mode water [STMW], central mode water, and North Pacific intermediate water [NPIW]) with distinct microbial communities were identified, explaining ~11% of microbial variation beyond depth and geography, with biomarker taxa identified (e.g., *Actinomarinales* for STMW, *Nitrosopumilales* for NPIW). This study reveals the extent of Kuroshio–Oyashio influence on microbial communities and highlights the integrated impacts of large-scale currents and fine-scale water masses on shaping microbial biogeography in the KOE region.

**IMPORTANCE:**

The convergence of the Kuroshio and Oyashio currents shapes high microbial diversity, as well as complex microbial-mediated biogeochemical processes. However, investigations into the microbial distribution patterns in relation to these current systems remain limited in spatial resolution. This study with high-resolution samples reveals the extent of Kuroshio–Oyashio influence on microbial communities and advances the understanding of how multi-scale oceanographic processes influence microbial biogeographical patterns. It provides a fine-scale perspective for exploring microbial distribution and assembly in highly dynamic oceanic environments.

## INTRODUCTION

The Kuroshio and the Oyashio are two important currents in the North Pacific current system, each with distinct hydrological characteristics, that together form a dynamic and ecologically significant region where they meet (1). This confluence zone, known as the Kuroshio–Oyashio Extension (KOE) region, is characterized by intense physical and biogeochemical gradients. The two currents play vital roles in shaping marine ecosystems, influencing ocean circulation, nutrient distribution, and marine biodiversity.

Kuroshio is a powerful western boundary current that originates in the Western Pacific (2). Upon encountering the Oyashio, the Kuroshio merges into the eastward-flowing North Pacific Current (3). As the second-largest warm current in the world, Kuroshio transports a substantial amount of heat, inorganic nutrients, and chemical substances from low to mid-latitudes, playing a crucial role in sustaining biological productivity within the subpolar gyre (4–6). In contrast, Oyashio is a cold current in the subpolar gyre of the North Pacific (7). Around 40°N, off northeastern Honshu, Oyashio meets the Kuroshio, with some of its flow turning eastward into the North Pacific Current, while another portion subducts beneath the Kuroshio, forming a deep current (7). Oyashio transports cold, low-salinity, nutrient-rich water, supporting high biological productivity, particularly phytoplankton and zooplankton, which sustain abundant fisheries (8). The convergence of the two distinct currents forms the KOE region, causing the complex biogeochemical processes in the Western Pacific (9, 10).

Marine microorganisms are a crucial component in the ocean, widely participating in biogeochemical cycles. The high sensitivity to environmental changes and short generation time enable microorganisms to rapidly respond to environmental changes, changing the community structure and functional traits. As a result, microbial communities often exhibit pronounced spatiotemporal heterogeneity, which is particularly evident in the complex and dynamic KOE region (7). Previous studies have shown distinct differences in surface bacterial community composition between the Kuroshio, Oyashio, and their confluence region. The dominant microbial groups in the surface waters of the Kuroshio Extension (KE) include *Cyanobacteria* and *Actinobacteria*, though their relative abundances vary significantly between the Kuroshio and the confluence region (11–13). In contrast, the Oyashio source waters are primarily inhabited by *Proteobacteria* and *Bacteroidota*, and the transition from the East Kamchatka Current to the Oyashio has been found to reshape microbial community composition and metabolic functions (14). Furthermore, microbial preferences for long-chain versus short-chain n-alkanes significantly impact the microbial community structure in the surface waters of KE (15, 16). However, most studies have focused on the microbial communities in surface, upper-layer waters, or specific depths; the research on the fine-scaled vertical variations of microbial communities in the KOE region remains limited.

Furthermore, the ocean dynamics driven by the confluence of the Kuroshio and Oyashio currents extend far beyond the large-scale currents themselves. Capturing the mesoscale processes and water masses in the KOE region is also crucial for resolving the spatial patterns of microbial communities. The complex frontal structures, diverse water masses, and abundant mesoscale eddies driven by intricate oceanic dynamics are the main features of the KOE region (17–19). The KOE region is a key connection for mid-depth and deep ocean circulation in the North Pacific, contributing to the formation of various mode waters, such as subtropical mode water (STMW), central mode water (CMW), and Okhotsk Sea mode water. Another significant water mass, the North Pacific intermediate water (NPIW), also originates in this region (20–23). Once formed, these water masses are transported to subsurface layers, playing crucial roles in ocean circulation, climate regulation, and biogeochemical cycles, such as carbon cycling, serving as long-term indicators of climate change (18).

Several studies demonstrated that the water masses defined by unique physicochemical characteristics and circulation dynamics may contribute to interpreting microbial biogeography. For instance, the water masses in the KOE region significantly changed the concentration and composition of dissolved organic matter and controlled methane cycling, which may cause the variations of microbial composition (24, 25). Water mass boundaries could act as barriers to the dispersal of free-living microbes, leading to distinct community structures (26–28). Together, these findings indicate that the water masses may act as important factors that structure microbial community composition and distribution patterns across the KOE region. However, due to the challenges of sample collection, it is not easy to investigate microbial communities at the water-mass scale with a high-resolution data set. As a result, the microbial biogeographic patterns in the KOE region remain resolved at large-scale resolution. And the impact of physical oceanographic processes, such as water mass isolation and eddy-driven mixing, on microbial community structures in this region lacks a comprehensive, systematic investigation.

Therefore, seawater samples from six sites along the D transect in the KOE region were collected from May to June in 2021, with high-resolution vertical gradient sampling conducted from the surface to the bottom at 18–24 layers each site. The microbial communities were then analyzed using 16S rRNA amplicon sequencing, and the currents and water masses were finely resolved based on environmental parameters. This study aimed to capture the fine-scaled geographical distribution pattern of microbial communities in the KOE region and explore the multiple driving factors of large-scale currents and water masses in this study. It provides further insights into the fine-scale biogeographic patterns of microbial communities in the KOE region and sheds light on the influence of Kuroshio–Oyashio currents and water masses on microbial communities.

## RESULTS

### General physicochemical characterization and boundaries of Kuroshio and Oyashio currents

**Fig 1:**
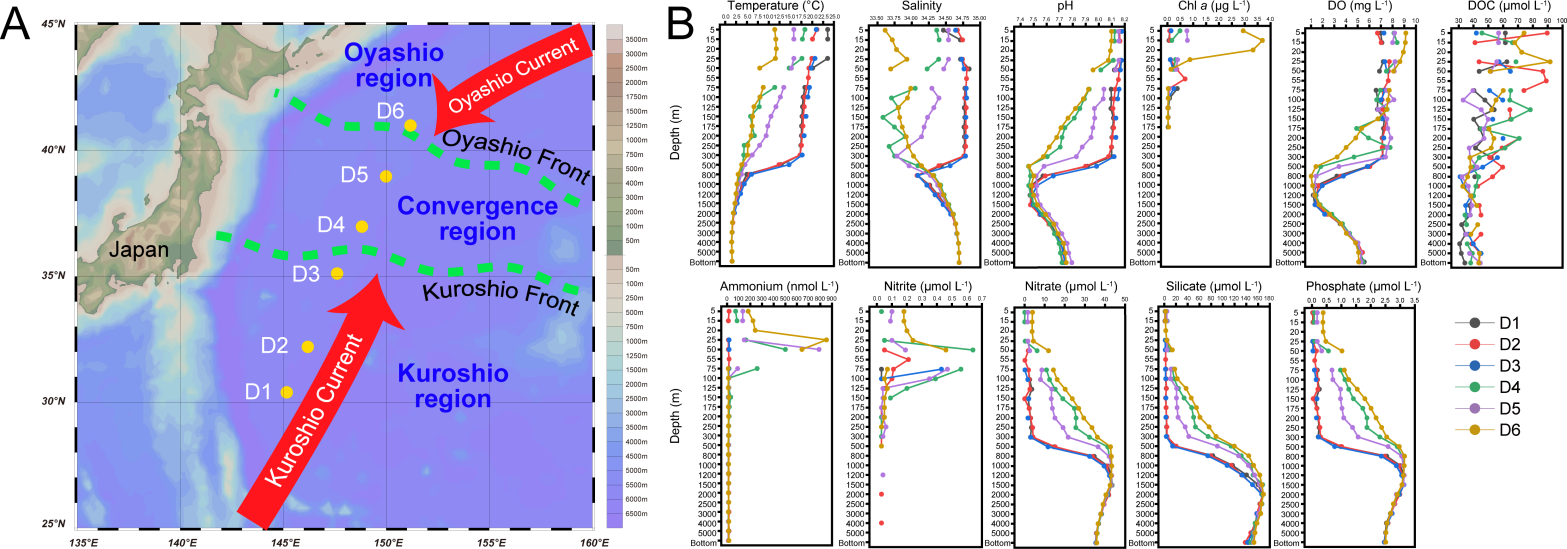
KOE region and vertical profiles of environmental parameters. (**A**) Map showing the location of D transect (sites D1–D6, yellow dots). The range of Kuroshio and Oyashio currents. Yellow dots represent sampling points, red arrows indicate ocean currents, and green dashed lines represent oceanic fronts. (**B**) The vertical profiles of environmental parameters. DO, dissolved oxygen; DOC, dissolved organic carbon.

The physicochemical parameters along the D transect display significant variations with both depth and latitude (Fig. 1B). The environmental parameters are highly consistent among sites D1–D3, with a pronounced thermocline around 500 m depth. Above the thermocline, relatively higher water temperature (17.20°C–23.55°C), salinity (34.46–34.84), and pH (8.07–8.18) were observed, with stable dissolved oxygen (DO) and low nutrient concentrations, reflecting the influence of warm and oligotrophic Kuroshio current. Below the thermocline, temperature declined rapidly (1.47°C–6.04°C), with NO_3_^−^, SiO_3_^2−^, and PO_4_^3−^ concentrations increasing markedly. Notably, pH (7.47–7.74), DO (1.18–5.56 mg L^−1^), and salinity (34.08–34.69) decreased sharply around the thermocline and gradually recovered with depth.

In contrast, no significant thermocline was found among sites D4–D6. These sites exhibit lower temperatures, salinity, and pH, as well as higher nutrient concentrations compared to D1–D3 at the same depths. The parameters show notable differences among D4–D6 in the upper 500 m, with a clear south-to-north gradient of decreasing eutrophication. Moreover, the physicochemical parameters tend to converge across the D transect at depths below 800 m.

To investigate the microbial distinctions in Kuroshio–Oyashio interactions, the samples are classified based on water characteristics. First, the positions of the Kuroshio and Oyashio fronts are identified with conductivity‐temperature‐depth (CTD) data, then defining the Kuroshio region (KR), Oyashio region (OR), and their confluence region (CR) (Fig. 1A). The Oyashio front is defined by a temperature of 5°C–8°C at a depth of 100 m, and the Kuroshio front is defined by a temperature of 14°C–15°C at 200 m depth (18). Sites D1–D3, located south of the Kuroshio front, are classified as the KR, mainly influenced by the warm, salty, and oligotrophic Kuroshio water. Site D6, located at the Oyashio front, is classified as the OR, primarily influenced by the cold, salty, and nutrient-rich Oyashio water. Sites D4 and D5 are located between the Kuroshio front and Oyashio fronts, representing the CR, where the combined effects of both currents are more pronounced than those of either current alone (18).

### Microbial abundance in the KOE region

**Fig 2:**
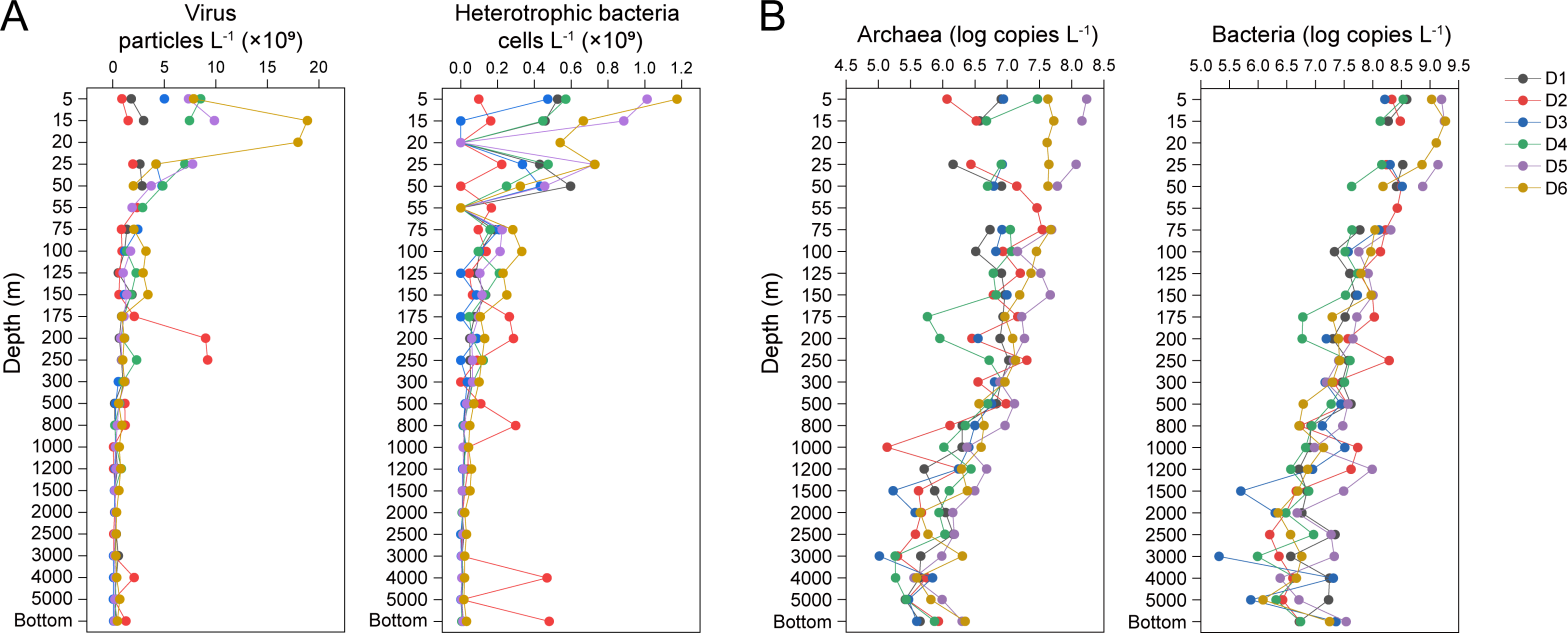
The abundance of microbial components along the D transect in the KOE region. (**A**) Heterotrophic bacterial and viral particle abundances (number per liter). (**B**) Abundances of archaeal and bacterial 16S rRNA gene copies (copies per liter).

Flow cytometric analysis shows that heterotrophic bacteria abundance in the D transect ranges from 1.00 × 10⁶ to 1.17 × 10⁹ cells L^−1^, with an average of 1.52 × 10⁸ cells L^−1^. Virus abundance ranges from 6.58 × 10⁷ to 1.89 × 10¹⁰ particles L^−1^, with an average of 1.81 × 10⁹ particles L^−1^ (Fig. 2A). Both components decrease gradually along depths, with the virus abundance peaks at 15–25 m depth and heterotrophic bacteria abundance peaks at 5 m. In contrast to other sites, the heterotrophic bacteria abundance and virus particle count of site D2 exhibit no clear trend.

Quantitative PCR (qPCR) analysis reveals depth-related decrease in bacterial and archaeal 16S rRNA gene abundances (Fig. 2B), which is consistent with the flow cytometric results. Bacterial 16S rRNA gene abundance ranges between 2.07 × 10⁵ and 1.85 × 10⁹ copies L^−1^, while archaeal 16S rRNA gene abundance ranges from 1.04 × 10⁵ to 1.71 × 10⁸ copies L^−1^. The abundance of bacteria is, on average, one order of magnitude higher than archaea.

In the horizontal gradient, the differences in heterotrophic bacterial and viral abundances across sites are primarily observed above 55 m. Notably, D5 and D6 (the Oyashio and convergence region) have significantly higher abundances than those in the Kuroshio region, according to the flow cytometric measurements (Kruskal–Wallis test, *P* < 0.001).

### Distinct regional and vertical variations of microbial diversity patterns

**Fig 3:**
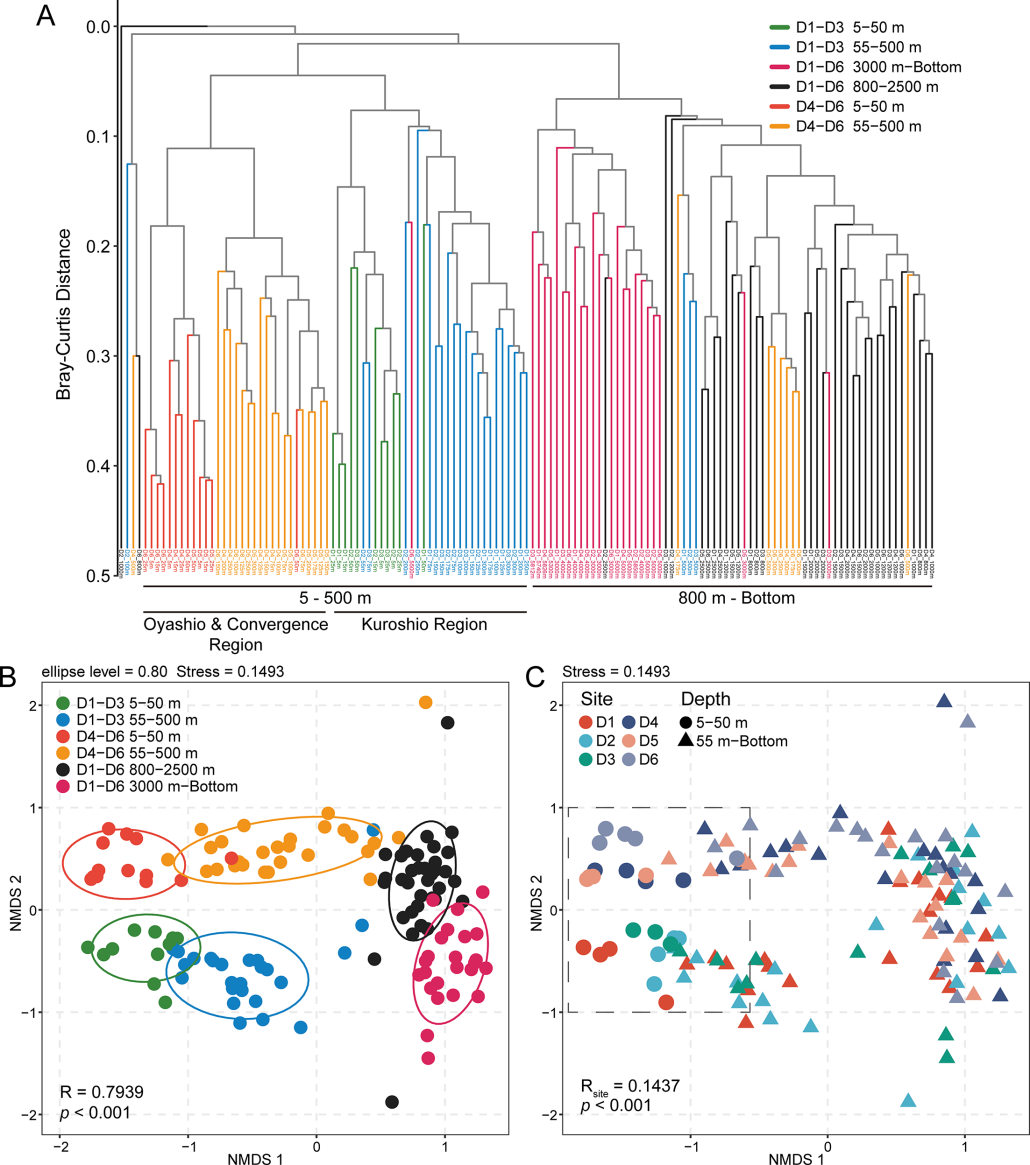
Hierarchical clustering and NMDS ordination of microbial community along the D transect in the KOE region. (**A**) Hierarchical clustering analysis. (**B**) NMDS ordination of microbial community based on Bray–Curtis dissimilarity. Samples are colored by site and depth groups. Ellipses represent 80% confidence intervals around group centroids. (**C**) Detailed view of the upper layer (5–50 m), with samples colored by individual site to highlight the distinct community compositions.

To investigate the variations of microbial communities in the KOE region, hierarchical clustering and non-metric multidimensional scaling (NMDS) analysis were conducted. Samples are primarily clustered with water depth, with 500 m as the first key transition depths (Fig. 3A). Furthermore, the Kuroshio Current has significant impact on microbial community structure within the upper 500 m layer, where the samples from Kuroshio region (D1–D3) are clearly separated from Oyashio and convergence region ones (D4–D6) (ANOSIM, R = 0.62, *P* < 0.001) (Fig. 3A and B). Notably, a more pronounced regional differentiation in microbial communities was observed within the 5–50 m depth layer, with distinct clustering among sites D1, D2–D3, D4–D5, and D6, indicating the combined influence of the Kuroshio and Oyashio Currents on the surface microbial community structure (Fig. 3C). In contrast, no site-specific clustering across D1–D6 is observed below 800 m depth. Meanwhile, several samples from 500 m and shallower depths got clustered with deep-water samples, particularly in the D4–D6 regions, while greater dissimilarities were observed between waters above and below 500 m in D1–D3 regions (ANOSIM, R_D1–D3_ = 0.82; R_D4–D6_ = 0.70).

Alpha diversity indices (Shannon, Simpson, and observed amplicon sequence variants [ASV]) reveal that species richness and diversity varied significantly with depths rather than across current regions (Fig. S1). Diversity is significantly lower within the 5–50 m depth layer compared to the 55–500 m layer (Kruskal–Wallis test, Shannon and observed ASV: *P* < 0.001; Simpson: *P* < 0.01). In contrast, no significant differences were found within the same layer among regions influenced by different currents. Corroborating this pattern, Pielou’s evenness index was significantly lower within the 5–50 m layer (Fig. S1D, Kruskal–Wallis test, *P* < 0.01), while higher values were observed in the 55–500 m layers, revealing dominance by a few highly abundant taxa in surface waters and a more even species distribution at intermediate depths.

### Spatial variations of microbial community compositions

**Fig 4:**
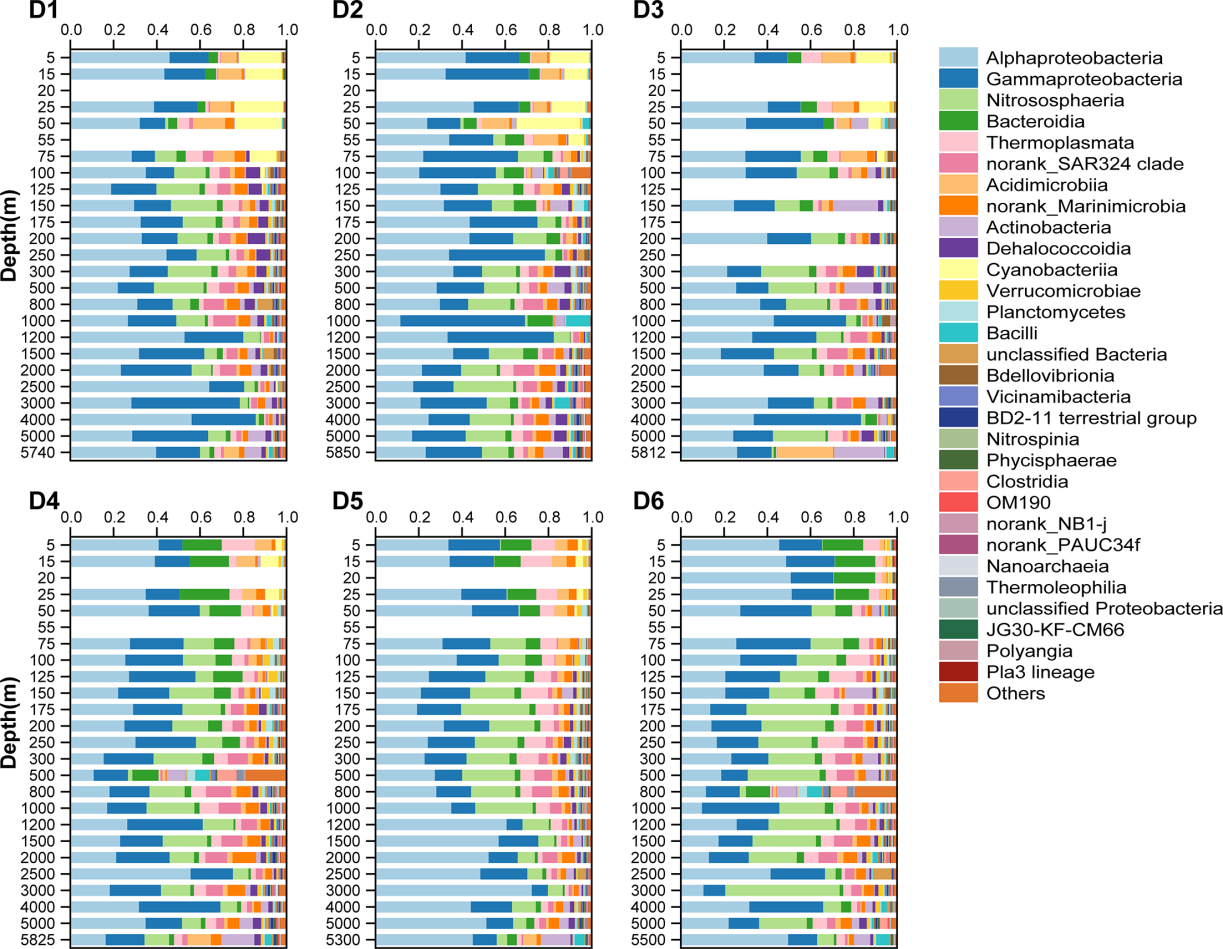
The microbial community compositions along the D transect in the KOE region. Top 30 abundant taxa at the class level are displayed, while the remaining taxa are grouped as “Others.” For each site, 18–24 depth layers from the surface to the bottom are shown.

Microbial community compositions along the D transect display clear regional and vertical variations (Fig. 4; Fig. S2). Overall, *Alphaproteobacteria* and *Gammaproteobacteria* are the dominant groups, together accounting for approximately 20%–45% of the total community across all depths. In the surface layers (5–50 m), communities of D1–D3 (the Kuroshio region) are clearly distinct from those of D4–D6 (the confluence and Oyashio regions). The former is characterized by elevated abundances of *Acidimicrobiia* and *Cyanobacteriia*, whereas the latter shows substantially reduced proportions of *Cyanobacteriia* and higher contributions of *Thermoplasmata* (mainly represented by Marine Group II) and *Bacteroidia* (mainly represented by *Flavobacteriales*), reflecting the more eutrophic conditions of the Oyashio current. Within these layers, D4–D5 still retain certain contributions of *Cyanobacteriia*, whereas D6 is almost completely dominated by heterotrophic groups, indicating a stronger influence of the Oyashio current in D6 surface.

In the 55–500 m layers, *Nitrososphaeria* (mainly represented by *Nitrosopumilales*), *Thermoplasmata*, and SRA324 gradually increase in relative abundance, while *Cyanobacteriia* and *Bacteroidia* markedly decrease. Compared with D1–D3, D4–D6 exhibit higher relative abundances of *Nitrososphaeria*, *Thiomicrospirales*, and *Flavobacteriales*.

In the deep layers (>800 m), microbial communities are dominated by *Alphaproteobacteria*, *Gammaproteobacteria,* and *Nitrososphaeria*, with *Marinimicrobia* (SAR406 clade) showing a consistent increase. In contrast to the upper layers, the influence of the two currents diminishes, resulting in community structures that are largely site-specific rather than current-driven. For instance, *Alphaproteobacteria* is relatively abundant at D5, whereas *Nitrososphaeria* predominates at D6.

### Environmental correlations and microbial interactions

**Fig 5:**
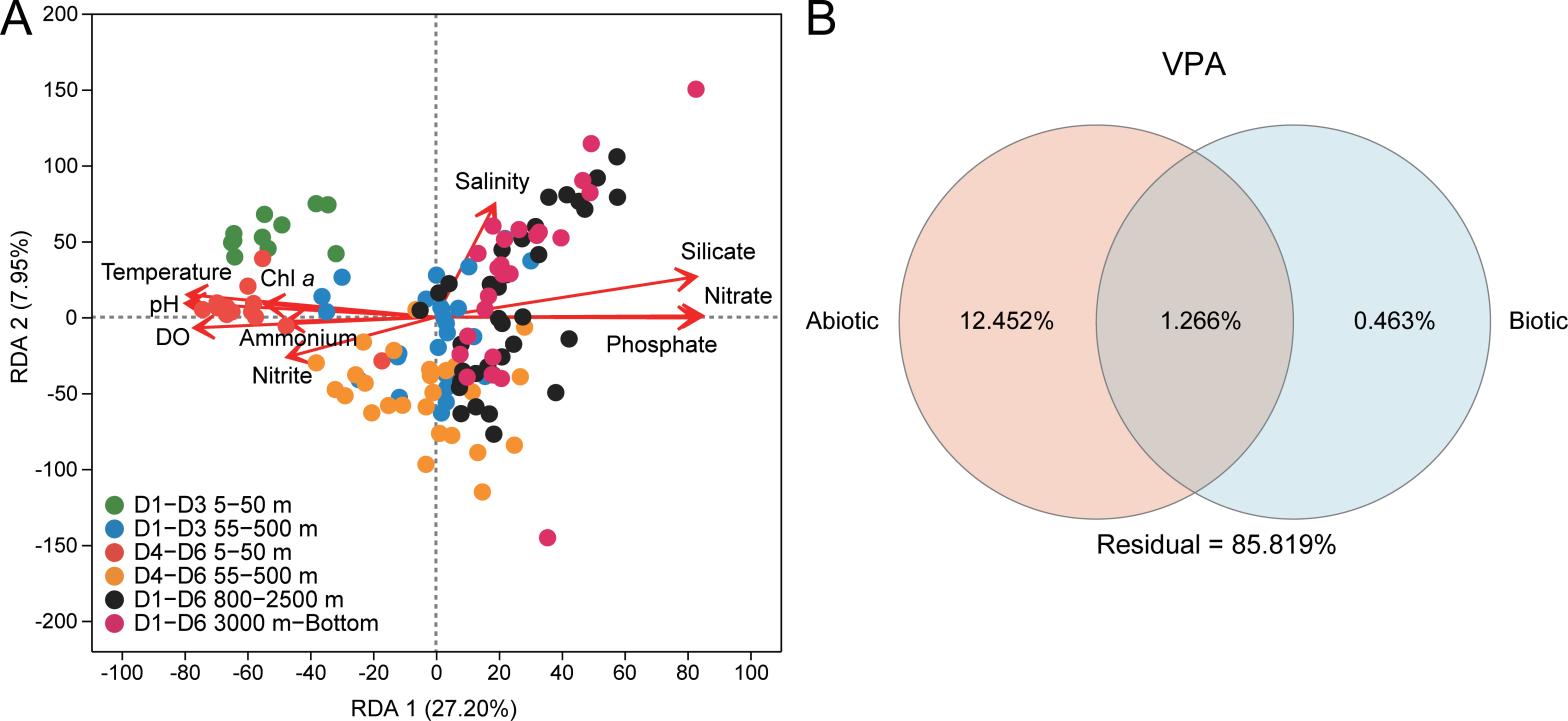
Correlations between environmental parameters and microbial communities in the D transect of the KOE region. (**A**) RDA showing the relationships between microbial community structures and environmental parameters. (**B**) Variance partitioning analysis (VPA) illustrating the proportion of community variation explained by abiotic (temperature, salinity, DO, pH, NH₄^+^, NO₃⁻, PO₄³⁻, SiO₃²⁻, chlorophyll *a*, and dissolved organic carbon [DOC]) and biotic (abundances of bacteria and archaea) factors, and their shared effects.

Redundancy analysis (RDA) shows that the spatial differentiation of microbial communities along the D transect is strongly correlated with multiple environmental parameters. Among the measured parameters, temperature, DO, and inorganic nutrients are the main correlated ones with the depth-related variations, leading to distinct separation of samples along the RDA1 axis, with stronger effects on communities above 500 m. As for the separation among the Kuroshio, confluence, and Oyashio regions, salinity is the main contributing factor (Fig. 5A). Among water layers, the environmental parameters that strongly correlated with microbial community variation differ. In the surface (5–50 m), NH₄^+^, DO, and temperature show the strongest correlations with community variations. Below 800 m, salinity exhibits the highest correlation among all measured parameters, followed by temperature and SiO_3_^2−^ above 2,500 m, and PO_4_^3−^, NO_3_^−^, and DO between 3,000 m and the bottom. Notably, microbial communities in the intermediate layer (55–500 m) show significant correlations with multiple factors, including nutrients, temperature, pH, and salinity (Fig. S3).

**Fig 6:**
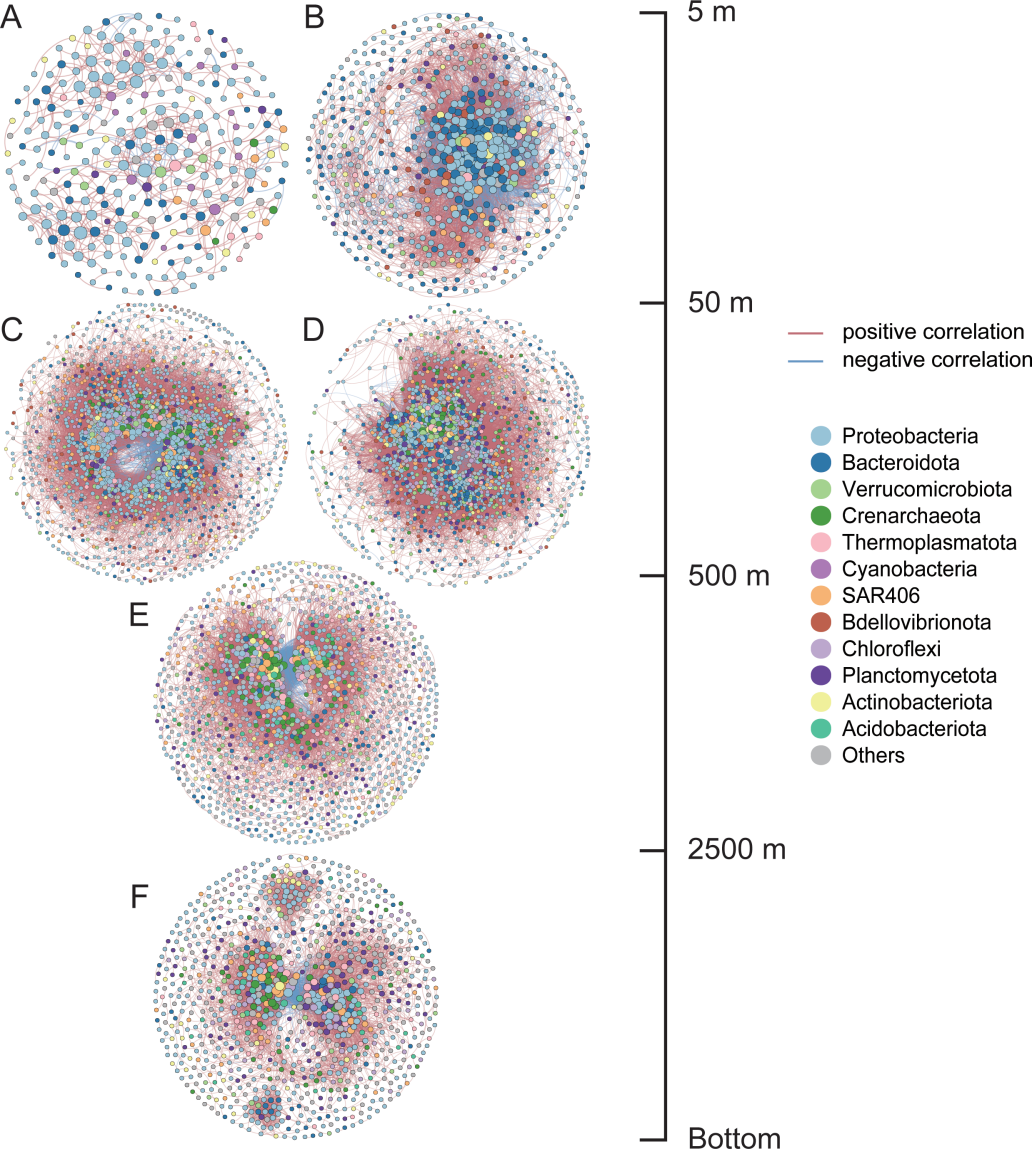
Co-occurrence networks of microbial community in different regions in the D transect of the KOE region. Networks were constructed at the ASV level based on Spearman’s correlations (|ρ| > 0.7, *P* < 0.01), including ASVs present in more than 20% of samples. (**A**) 5–50 m at sites D1–D3; (**B**) 5–50 m at sites D4–D6; (**C**) 55–500 m at sites D1–D3; (**D**) 55–500 m at sites D4–D6; (**E**) 800–2,500 m at sites D1–D6; (**F**) 3,000 m bottom at sites D1–D6.

Interactions among microorganisms could also shape microbial community structure. Co-occurrence network analysis reveals strong modularity of microbial communities of D transect, with *Proteobacteria*, *Crenarchaeota*, and *Bacteroidota* serving as key taxa (Fig. S4). Moreover, network structures differ across current regions and depths (Fig. 6, Table S1). The surface networks at sites D1–D3 are relatively simple, with fewer nodes and significant associations than other regions, whereas those at D4–D6 are more complex, with *Bacteroidota* accounting for 23.56% of nodes. This implies that the Kuroshio-influenced surface waters are characterized by relatively stable conditions, where a few taxa dominate the communities, resulting in a more constrained community structure. The 55–500 m layer exhibits the highest complexity (D1–D3: 1,935 nodes, 76,942 edges; D4–D6: 1,779 nodes, 104,154 edges), where positive edges comprise 91% and 77% of correlations, respectively. The network at the intermediate layer exhibited higher connectivity compared with deeper layers. *Thermoplasmatota* and *Planctomycetota* contribute more nodes in the D4–D6 networks, showing their important role in maintaining microbial network stability. In the deep layers, networks progressively simplify with increasing depth, showing shifts in microbial interaction patterns across different depths and current-influenced regions.

Overall, environmental parameters together with depth explain 13.72% of the community variation, and biotic factors account for 1.73%, leaving 85.82% unexplained (Fig. 5B).

### Water mass stratification and biomarker microbial taxa

**Fig 7:**
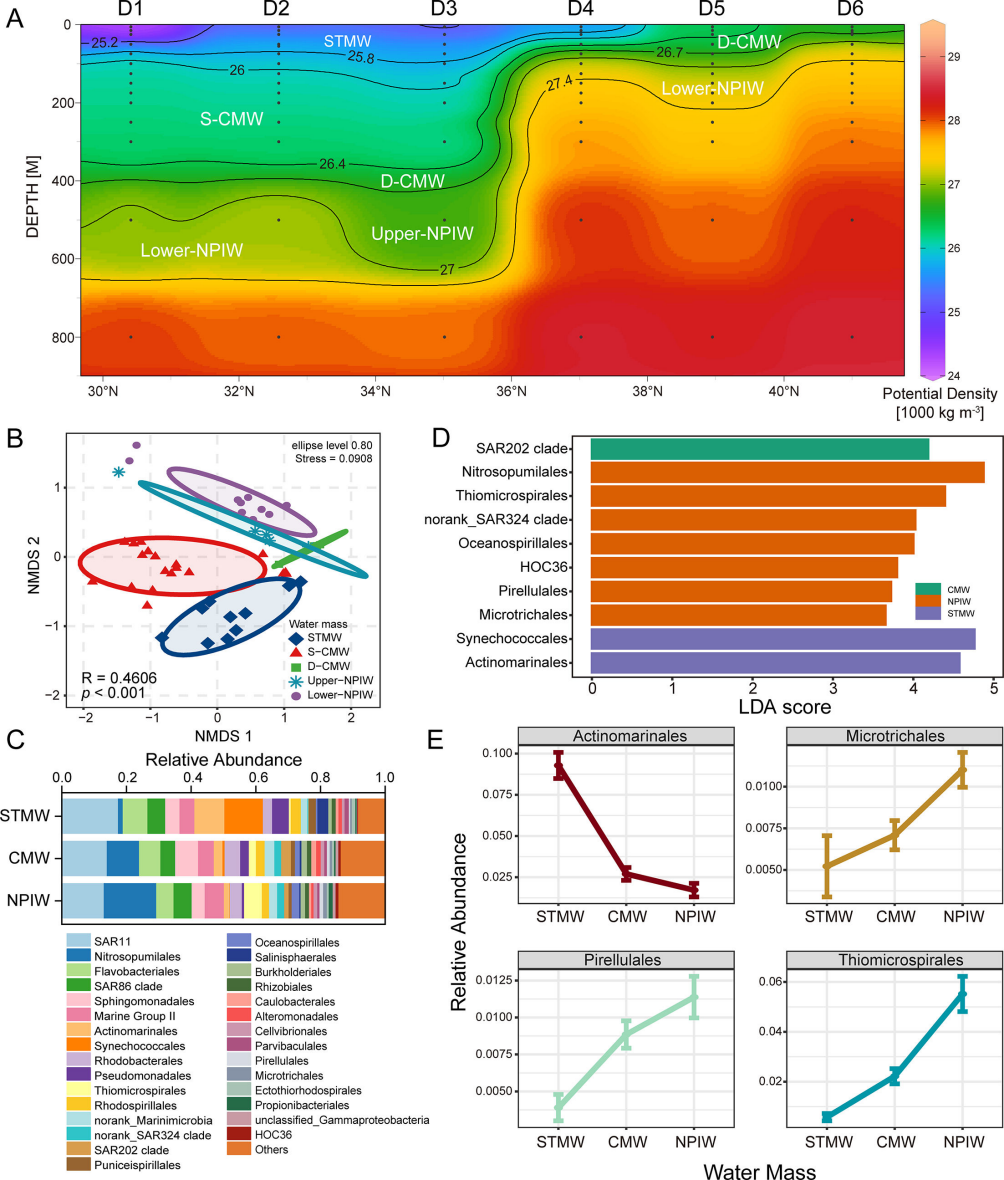
Analyses of microbial communities in water masses. (**A**) Vertical section along D transect showing the distribution of water masses defined by their potential density ranges. The region was distinguished as subtropical mode water (25.2–25.8 σ_θ_), shallow part of the central mode water (26.0–26.4 σ_θ_), dense part of central mode water (26.4–26.7 σ_θ_), upper North Pacific intermediate water (26.7–27.0 σ_θ_), and lower North Pacific intermediate water (27.0–27.4 σ_θ_). The map was created using Ocean Data View version 5.1.2 (https://odv.awi.de). (**B**) NMDS analysis of microbial community in different water masses of KE. (**C**) The microbial community compositions in three water masses of KE. (**D**) Biomarkers in three water masses identified by linear discriminant analysis effect size (LEfSe) analysis. (**E**) Relative abundance of microbial taxa identified as water-mass indicators based on low within-water-mass coefficient of variation (CV < 0.7) and significant Kruskal–Wallis test (*P* < 0.05).

Beyond the influences of common physical and chemical parameters and microbial interactions, the unique and dynamic hydrodynamic conditions of the KOE region also play a role in shaping microbial communities. To further investigate the unexplained variation in microbial community composition, water masses were additionally stratified based on potential density. Three major water masses were identified in the shallow and intermediate layers along the D transect: STMW (25.2–25.8 σ_θ_), CMW, further subdivided into shallow (S-CMW, 26.0–26.4 σ_θ_) and dense (D-CMW, 26.4–26.7 σ_θ_) layers, and NPIW, subdivided into upper NPIW (26.7–27.0 σ_θ_, originating from the Sea of Okhotsk) and lower NPIW (27.0–27.4 σ_θ_, influenced by subtropical circulation) (Fig. 7A) (18). Above all, STMW was mainly distributed above 75 m at sites D1–D3 and above 15 m at D4. CMW occurred at 100–300 m at D1–D3, 50 m at D4, <25 m at D5, and <20 m at D6. NPIW was detected at ~500 m at D1–D3, 75–125 m at D4, 75–200 m at D5, and 25–75 m at D6 (Table S2).

NMDS reveals clear separation of microbial communities in different water masses, suggesting the strong structuring effect of water masses on microbial communities (Fig. 7B, R = 0.461, *P* < 0.001). Although water-mass distribution is closely associated with depth and site, variance partitioning indicates that water masses independently explained ~11% of microbial community variation beyond the effects of depth and geography, highlighting the structuring role of the special water masses in the KOE region on microbial community (Fig. S5). VPA further revealed a shared fraction between water masses and physicochemical parameters (10.6%), suggesting that the influence of water masses mainly results from their aggregation of distinct physicochemical conditions. Overall, microbial community composition gradually shifts across STMW, CMW, and NPIW water masses. The relative abundances of *Synechococcales*, *Flavobacteriales*, *Actinomarinales*, *Pseudomonadales*, and *Salinisphaerales* decrease from STMW to CMW and NPIW, whereas *Nitrosopumilales*, Marine Group II, *Thiomicrospirales*, and the SAR324 clade exhibit the opposite trend (Fig. 7C). When further stratifying water masses based on potential density, some taxa display considerable variation across subregions within the same water mass (Fig. S6). For instance, the SAR11 clade shows low relative abundance in S-CMW but reaches its highest relative abundance in D-CMW across all water masses, suggesting a combined influence of water mass and other environmental factors.

Nevertheless, certain taxa remain stable within a given water mass and differ significantly among water masses. Based on the LEfSe analysis (linear discriminant analysis [LDA] score > 3.5, *P* < 0.05) combined with CV and Kruskal–Wallis significance, *Thiomicrospirales* and *Pirellulales* are identified as biomarkers of NPIW, whereas *Actinomarinales* is a biomarker of STMW (Fig. 7D and E). Other taxa, such as *Nitrosopumilales*, SAR202 clade, and *Synechococcales*, are enriched within individual water masses but exhibited variable abundances across subregions, reflecting the influence of additional environmental factors.

## DISCUSSION

With high-resolution and multi-layered samples, the coordinated variation of environmental parameters and microbial communities under the influence of the Kuroshio and Oyashio were observed, clarifying the horizontal and vertical extent of the Kuroshio–Oyashio influence on microbial communities. In addition, the structuring role of water masses in shaping these communities was captured. This study provides a high-resolution perspective for understanding how hydrographic currents, water mass characteristics, and environmental gradients collectively shape microbial biogeography, revealing patterns of community distribution and ecological adaptation in this special oceanic region.

### High-resolution sampling delineates the boundaries of Kuroshio–Oyashio influence on microbial communities

With high-resolution data, the influence of the Kuroshio–Oyashio confluence was most pronounced within the upper 500 m, where coordinated variations in environmental parameters, microbial abundance, diversity, community composition, and interactions were observed. Environmental parameters, including temperature, salinity, pH, and concentrations of inorganic nutrients, exhibited significant spatial heterogeneity above 500 m. The Kuroshio region was characterized by warm, high salinity, and oligotrophic conditions, whereas the Oyashio and confluence regions were cold, low salinity, and nutrient-rich (18). Correspondingly, microbial communities displayed clear regional differences: the Kuroshio region harbored lower abundance, consistent with oligotrophic conditions, while the Oyashio and confluence regions supported higher abundance and diversity with more complex community interactions, reflecting the influence of nutrient-rich currents.

Firstly, differences between Kuroshio- and Oyashio-influenced waters were especially marked in the upper 50 m, consistent with a previous study on surface prokaryotes (29). In the Kuroshio region (D1–D3), photoautotrophic and oligotrophic-adapted microbes (e.g., SAR11 clade and *Cyanobacteria*) were enriched, reflecting adaptation to warm and oligotrophic euphotic conditions. By contrast, eutrophic taxa (e.g., *Bacteroidota*) were more abundant in the Oyashio and confluence regions, particularly at the Oyashio-dominated site D6. These patterns were likely shaped not only by light availability and nutrient concentrations but also by variations in phytoplankton (30, 31), eukaryotic microbial communities (14, 32), and algal blooms (33). The analyses in our study indicated that ammonium and DO concentrations were key environmental drivers of surface microbial variations, which jointly contributed to microbial community assembly in surface waters.

In the 50–500 m layer, microbial diversity increased, accompanied by gradual changes in SiO_3_^2−^, PO_4_^3−^, NO_3_^−^, and temperature. The relative abundance of *Nitrosopumilales*, *Thermoplasmata*, and the SAR324 clade increased, suggesting enhanced nitrification and chemolithoautotrophic processes in this depth range (34, 35). On the other hand, the co-occurrence network revealed more complex microbial interactions in 50–500 m compared to surface and deep layers. Li et al. compared the co-occurrence properties across the Kuroshio South of Japan, KE, and the Kuroshio–Oyashio interfrontal zone, revealing the stability of microbial networks (36). Building on this, based on higher-depth resolution samples, our study compared co-occurrence networks across different depths and regions, showing the shift of microbial interactions. The higher proportion of positive correlations in the Kuroshio region suggests microbial cooperation to adapt to environmental changes, whereas negative correlations are more prevalent in the Oyashio and confluence regions, reflecting competition and ecological niche differentiation in this region (37, 38).

At approximately 500 m, a sharp transition in multiple environmental parameters was observed, marking the boundary of Kuroshio–Oyashio influence. The transition was particularly pronounced in the Kuroshio region, with rapid decreases in temperature, salinity, pH, and DO, and concurrent increases in SiO_3_^2−^, PO_4_^3−^, and NO_3_^3−^, highlighting a distinct physical and chemical boundary. NMDS analysis revealed corresponding shifts in microbial community structure across this depth, indicating that the 500 m transition represents both a physical-chemical boundary and the depth limit of Kuroshio–Oyashio influence on microbial communities.

Below 800 m, the stable environmental parameters and microbial community structure indicated minimal influence of surface current interactions. Beta diversity analysis revealed no significant differences among sites in deep waters, but clear vertical stratification. Meanwhile, microbial co-occurrence networks became less complex, indicating reduced microbial interactions under the relatively stable and low-energy conditions of the deep sea (36). Although pressure was not measured in this study, it is likely one of the key factors controlling microbial community composition in deeper layers.

Overall, high-resolution, multi-layered sampling delineated the vertical and horizontal boundaries of Kuroshio–Oyashio influence on microbial communities, providing insights into community distribution patterns, ecological adaptation, and the role of environmental gradients in shaping microbial biogeography in this special region. It should be noted that the hydrographic characteristics of this region exhibit pronounced seasonal and interannual dynamics. Our study is only based on high-resolution samples collected from May to June 2021; however, the comprehensive analyses are essential to explore the spatiotemporal patterns of the horizontal and vertical extents of Kuroshio–Oyashio currents and associated water masses in other years and seasons.

### Distinct water masses explain microbial community variation beyond depth and geography

RDA and VPA indicated that the measured physicochemical and spatial parameters in this study explained only a small fraction of the observed microbial community variations, with 85.8% remaining unexplained. Similarly, low explanatory power of environmental parameters has been reported in other studies carried out along a Kuroshio transect, showing weak correlations between surface-layer microbial richness and environmental parameters (39). Such limitations may arise from the parameters measuring restriction, ignorance of biotic effects (40), and the inherent complexity of dynamic oceanic systems (41, 42). Other than the parameters measured in this study, multiple biotic and abiotic factors have been reported to vary in the KOE region, such as dissolved organic matter composition, trace metals, viral lysis, and grazing (24, 43, 44). These factors may also shape microbial community structures and further contribute to the unexplained variation.

In fact, the KOE region is shaped not only by large-scale currents but also by finer-scale oceanographic processes, such as water mass and mesoscale eddies. Several studies have demonstrated that the water masses defined by unique physicochemical characteristics and circulation dynamics may contribute to interpreting microbial biogeography. Furthermore, such water mass boundaries could constrain the dispersal of free-living microbes, leading to distinct community structures (26–28). To explore the spatial patterns of microbial community in this region, three water main masses were differentiated based on potential density (STMW, CMW, and NPIW). Our study indicates that during the sampling period in 2021, these water masses occupied markedly different depth ranges in the Kuroshio, Oyashio, and confluence regions.

Notably, it was found that water masses accounted for 11% of the microbial community variation beyond depth and geography. Variance partitioning revealed a notable shared fraction (10.6%) between water masses and physicochemical parameters, suggesting that water masses shape microbial communities by aggregating distinct physicochemical conditions. Moreover, several biomarkers were found for each water mass. STMW, formed in the winter mixed layer, is prominent in the Kuroshio recirculation area (45, 46). Analysis in this study reveals that *Synechococcales* and *Actinomarinales* are particularly enriched in STMW, with *Actinomarinales* identified as a biomarker of STMW. Strains of *Actinomarinales* have been reported to be aerobic photoheterotrophs and have streamlined genomes. They have the potential to utilize exogenous amino acids and cyanobacterial-derived organic compounds as carbon and energy sources, which may help with their adaptation to the STMW environment (47). CMW, formed north of KE, is a major sink for atmospheric CO_2_ and plays a key role in carbon sequestration (48, 49). Several taxa displayed varied relative abundance in S-CMW and D-CMW, indicating the complexity of water-mass dynamics and a combined influence of multiple factors. NPIW is formed by mixing low-salinity Oyashio and high-salinity KE intermediate waters (50). The potential biomarkers of NPIW (e.g., *Nitrosopumilales*, *Thiomicrospirales,* and *Pirellulales*) may reveal the chemoautotrophic feature of NPIW in deep-sea biogeochemical processes. As ammonia-oxidizing archaea, members of *Nitrosopumilales* have the ammonia oxidation and carbon fixation potential, linking nitrification to carbon fixation in intermediate NPIW waters (51). Similarly, members of *Thiomicrospirales* could function as sulfur-oxidizing chemolithoautotrophs, mediating sulfur cycling and inorganic carbon assimilation (52). Collectively, these taxa may contribute to the microbial carbon pump, thereby influencing long-term carbon storage in the KOE region (53). However, further analyses are required based on metagenomic sequencing to elucidate their ecological functions in this region.

This study, with a fine-scale approach, investigated microbial community variations across distinct water masses rather than focusing solely on geographic locations or depth layers. This perspective provides a more comprehensive perspective for analyzing and interpreting microbial spatial distribution patterns in the KOE region.

### Conclusion

High-resolution and multi-layered sampling in this study revealed the combined effects of hydrographic currents and water masses on microbial community variations in the KOE region. The KOE influence on microbial community variations extended from the surface down to 500 m, where temperature, salinity, pH, and inorganic nutrient concentrations displayed strong spatial heterogeneity. In the oligotrophic Kuroshio region, communities were enriched in photoautotrophic and oligotrophic-adapted taxa, whereas nutrient-rich Oyashio and confluence regions supported higher microbial abundances, greater taxonomic diversity, and more complex interaction networks with *Bacteroidota* and Marine Group II as key taxa. Below 500 m, microbial communities varied primarily with depth and exhibited no horizontal regional differentiation. Despite accounting for only a modest proportion of microbial variation, water mass stratification provided an additional dimension of variation beyond depth and geography, accounting for ~11% of observed variation. STMW, CMW, and NPIW exhibited variation in microbial composition, each containing characteristic microbial taxa, with *Actinomarinales*, *Nitrosopumilales*, and *Thiomicrospirales* identified as biomarkers. These findings highlight the important role of water masses in constraining microbial dispersal and shaping community differentiation. Overall, this study demonstrates that fine-scale, multi-layered sampling is essential to resolve the spatial heterogeneity of microbial communities in the KOE region and captures how currents and water masses jointly shape microbial communities. These results can provide new insights into microbial biogeography and ecological adaptation in one of the most dynamic oceanic environments of the North Pacific.

## MATERIALS AND METHODS

### Sample collection and environmental characterization

Seawater samples were collected from six sites (D1–D6) along the D transect (145.2°−152° E, 30.4°−42.4° N) in KOE region of the Northwest Pacific on board the research vessel “DongFangHong 3” (8 May to 25 June 2021) (Fig. 1). A total of 135 seawater samples were collected from the surface (5 m) to deep ocean (5,300–5,850 m) using Niskin bottles installed in a CTD rosette, from which seawater salinity and temperature were also recorded. At each site, 18–24 layers were sampled to capture fine-scale vertical variability. Detailed information on sampling sites and depths is provided in Table S3.

For DNA analyses, 1 L of seawater from each sample was filtered in sequence through 3 and 0.22 µm (47 mm, Millipore) pore size polycarbonate membranes. All filters were stored in liquid nitrogen onboard and transferred to −80°C until DNA extraction.

For flow cytometric analysis, 4 mL seawater samples were fixed with 0.5% glutaraldehyde (final concentration) for 15 min at room temperature, then stored in liquid nitrogen and transferred to −80°C until analysis (54). Three replicates were preserved for each sample.

Data on dissolved inorganic nutrients, DO, DOC, and chlorophyll *a* were provided by the Deep-Sea Layer and Earth System Frontier Science Center Laboratory, based on shared data from the cruise. For DOC, 40 mL water samples were immediately filtered through pre-combusted (at 450°C) 0.7 µm GF/F glass fiber filters (Whatman, UK) upon collection. The filtered samples were then stored in pre-combusted glass bottles at −20°C until further analysis. The DOC concentration was determined using a Shimadzu TOC-L CPH analyzer by the high-temperature (720°C) combustion catalytic oxidation method (55). The deep-sea water reference obtained from the Hansell Biogeochemical Laboratory at the University of Miami, USA, was utilized as a standard for measurement validation. For dissolved inorganic nutrients (nitrate, nitrite, silicate, and phosphate), seawaters were filtered through pre-combusted (450°C, 4 h) GF/F glass fiber filters (Whatman, UK) and stored at −20°C before being analyzed using a QuAAtro Analyzer (SEAL, Germany). Ammonium concentration samples were collected without filtration and determined by the fluorometric ammonium method on board immediately after collection (56, 57). The chlorophyll *a* samples were filtered through 0.7 µm GF/F glass fiber filters (Whatman, UK) and analyzed with a fluorescence spectrophotometer (Hitachi F4500) (58). DO was sampled and measured onboard following the Winkler method (59).

Potential temperature and potential density were calculated using CTD-derived depth, temperature, salinity, and pressure data and were used as the basis for the water mass division along the D transect. The calculation method is as follows (60).

#### Potential temperature

The definition of potential temperature is the temperature of a seawater mass moved adiabatically from an initial pressure (P) to a reference pressure ($P_{r}$) under constant salinity. It can be expressed as


$$\theta \left(S_{0},\;T_{0},\;P_{0},P_{r}\right)=T_{0}+\int_{P_{0}}^{P_{r}}\Gamma \left[S_{0},\;\theta \left(S_{0},\;T_{0},\;P_{0},\;P\right),P\right]\text{dP},$$

where Γ represents the adiabatic lapse rate.

#### Potential density

The potential density is defined as the density of a water mass moved adiabatically from an initial pressure to a reference pressure under constant salinity. Its definition can be written as


$$\sigma \left(S,\;T,\;P,\;P_{r}\right)=\rho \left[S,\;\theta \left(S_{0},\;T_{0},\;P,\;P_{r}\right),\;P\right]=\rho \left(S,\;\theta ,\;P\right).$$

Usually, potential density is expressed as ρ−1,000 $kg/m^{3}$, where ρ is density, with the same units but more convenient for use.

### DNA extraction, sequencing, and read processing

Total genomic DNA was extracted with the DNeasy PowerSoil Pro Kit (QIAGEN, Germany) following the instructions. Same amount of 3 and 0.22 µm filters from the same sample were cut into pieces and placed into the same sterile centrifuge tube. DNA quality and concentration were measured using the Nanodrop-2000 UV spectrophotometer (Thermo Fisher Scientific). Then the DNA was stored at −80°C before being sequenced.

For bacterial and archaeal 16S rRNA gene amplification, universal primers 515FmodF (5′-GTGYCAGCMGCCGCGGTAA-3′) and 806RmodR (5′-GGACTACNVGGGTWTCTAAT-3′) were used to target the V4 hypervariable region (61). The PCR reaction program was as follows: 95°C for 3 min; 29 cycles of 95°C for 30 s, 55°C for 30 s, and 72°C for 45 s; and a single extension at 72°C for 10 min. The amplifications were conducted in triplicate in a 20 µL mixture containing 0.4 µL FastPfu polymerase, 2 µL 2.5 mM dNTPs, 0.8 µL of each primer (5 µM), 4 µL 5× FastPfu buffer, and 10 ng template DNA. The PCR products were then purified using the AxyPrep DNA Gel Extraction Kit (AXYGEN) and quantified using the QuantiFluor-ST (Promega). Purified amplicons were paired-end sequenced in equimolar on the Illumina MiSeq PE300 platform (2 × 300) at Majorbio Bio-Pharm Technology Co., Ltd. (Shanghai, China). Raw reads are available at the National Omics Data Encyclopedia (NODE) database under accession number OEP00004846.

### Quantitative PCR and flow cytometry

Bacterial and archaeal 16S rRNA genes were quantified by qPCR with primers B967F (5′-CAACGCGAAGAACCTTACC-3′)/B1046R (5′-CGACAGCCATGCANCACCT-3′) and A967F (5′-AATTGGCGGGGGAGCAC-3′)/A1060R (5′-GGCCATGCACCWCCTCTC-3′), respectively (62, 63). The reaction system (20 µL) contained 10 µL of SYBR Premix Ex Taq II (2×, TaKaRa), 0.4 µL of ROX Reference Dye II (50×, TaKaRa), 0.4 µL (for bacteria), or 0.8 µL (for archaea) of each primer (10 µM), and 2 µL of template DNA.

The thermal cycling conditions for the bacterial 16S rRNA gene were 94°C for 3 min for pre-denaturation, followed by 35 cycles of 94°C for 30 s, 57°C for 45 s, and an extension at 72°C for 30 s. For the archaeal 16S rRNA gene, the steps consisted of 94°C for 3 min for pre-denaturation, 40 cycles of 94°C for 30 s, 50°C for 30 s, and an extension at 72°C for 30 s. All assays were conducted in triplicate, with negative controls. Standard curves were generated by amplifying plasmids carrying the corresponding PCR products. The amplification efficiency ranged between 90% and 110%, with R² >0.99, ensuring the reliability of the results.

For the abundances of virus and heterotrophic bacteria, triplicate samples were unfrozen to room temperature before analysis. For the virus, samples were diluted 100-fold with 0.02 µm filtered Tris-EDTA buffer (pH 8) and stained using SYBR green I (Thermo Fisher, USA) at 80°C for 10 min in the dark. For bacteria, samples were diluted 10-fold and stained at room temperature for 15 min in the dark. The incubated samples were cooled at room temperature and analyzed with a CytoFLEX flow cytometer (Beckman, Shanghai, China) (54).

### Data analysis and statistics

Raw reads from Illumina sequencing were first split by sample with barcodes. Low-quality reads and adaptors were removed by trimming raw reads using fastp (64). The reads were then assembled using FLASH based on the overlap between the paired-end reads (65), resulting in optimized data with 34,402,386 reads and an average length of 253 bp. The optimized data were processed using a denoising method with DADA2 to obtain ASVs (66). ASVs were assigned taxonomy against the SILVA database (release 138, http://www.arb-silva.de) with the RDP classifier (version 11.5, https://sourceforge.net/projects/rdp-classifier/) and a confidence threshold of 70% (67).

The alpha diversity indices of the microbial communities were calculated with Mothur (68). The Kruskal–Wallis non-parametric test was employed to compare differences in alpha diversity indices among different groups. For beta diversity, hierarchical clustering analysis and NMDS analyses were conducted based on the Bray–Curtis distance algorithm with the “vegan” package (version 2.4.3) in R (69). Spearman’s correlation analysis was used to assess the correlations between microbial communities and environmental parameters. In addition, canonical RDA and VPA were executed to analyze the impact of these factors on microbial communities with the “vegan” package (version 2.4.3) in R (69). Compositional data were Hellinger transformed, and environmental parameters were logarithmically transformed prior to the analyses. The LEfSe analysis was used to identify the biomarkers of different water masses with the “microeco” package (LDA score > 3.5) (70, 71). To further validate water-mass biomarkers, the CV for each taxon within water masses was calculated, and a Kruskal–Wallis test was performed. Taxa with low CV (CV < 0.7) and significant differences (*P* < 0.05) were considered stable biomarkers of water-mass-specific patterns.

To explore the microbial interactions in each region, the co-occurrence network at the ASV level was constructed for each group, respectively. ASVs present in more than 20% of the samples were selected to construct the network, with Spearman’s correlation coefficient |ρ| >0.7 and *P* value <0.01 were set to select valid interactions. Network conduction and topological parameter calculation were performed using R packages “igraph” (version 1.3.5) (72), “psych” (version 2.2.9), and “Hmisc” (version 4.7-1). Network visualization was performed with Gephi (version 0.9.7, https://gephi.org/) (73). All the analyses conducted in R were based on version 4.2.0 (74).

## Data Availability

Raw reads of all the high-throughput sequencing data in this study are available at the NODE database under accession number OEP00004846.

## SUPPLEMENTAL MATERIAL

Supplemental material aem.01960-25-s0001.pdf Figures S1 to S6; Tables S1 to S3.

ASM does not own the copyrights to Supplemental Material that may be linked to, or accessed through, an article. The authors have granted ASM a non-exclusive, world-wide license to publish the Supplemental Material files. Please contact the corresponding author directly for reuse.
