# Supplementary material for: From currents to water masses: fine-scale insights into microbial biogeography in the Kuroshio–Oyashio Extension region
Source: Appl Environ Microbiol. 2025 Dec 11;92(1):e01960-25. doi: 10.1128/aem.01960-25 (PMC12838210; doi:10.1128/aem.01960-25)
Supplement: Supplemental material — Figures S1 to S6; Tables S1 to S3. [file aem.01960-25-s0001.pdf]

Supplementary information

From currents to water masses: fine-scale insights into microbial biogeography in the Kuroshio-Oyashio Extension region

Rong Huang[1,2], Yulin Zhang[1], Lulu Han[2], Ronghua Liu[1], Xinyi Zhai[1], Ke Zeng[1], Guodong Song[3], Honghai Zhang[3], Peng Yao[3], Zhaohui Chen[5], Jiwen Liu[1,2,4] and Xiao-Hua Zhang[1,2,4]

[1]Frontiers Science Center for Deep Ocean Multispheres and Earth System, and College of Marine Life Sciences, Ocean University of China, Qingdao, China
[2]Laboratory for Marine Ecology and Environmental Science, Qingdao Marine Science and Technology Center, Qingdao, China
[3]Key Laboratory of Marine Chemistry Theory and Technology (Ministry of Education), Ocean University of China, Qingdao, China
[4]Key Laboratory of Evolution & Marine Biodiversity (Ministry of Education), and Institute of Evolution & Marine Biodiversity, Ocean University of China, Qingdao, China
[5]Key Laboratory of Physical Oceanography, Ministry of Education, Ocean University of China, Qingdao, China

Rong Huang and Yulin Zhang contributed equally to this work.

***Author for correspondence:**
Xiao-Hua Zhang, Email: xhzhang@ouc.edu.cn

Running title: Microbial biogeography in KOE region

This file includes:

Figures S1 to S6
Table S1 to S3

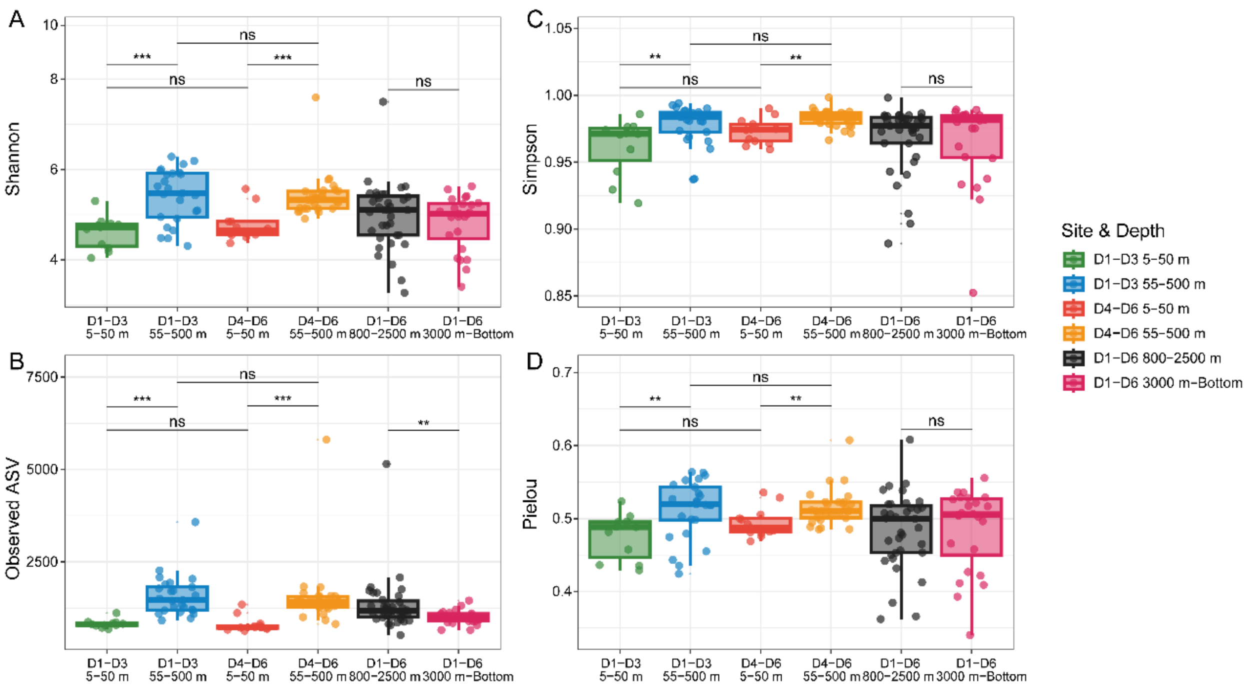

Fig. S1 The α-diversity of microbial community in D transect of the Kuroshio-Oyashio Extension (KOE) region. The Wilcoxon test between groups was marked by stars (***: $p < 0.001$, **: $p < 0.01$, ns: not significant). (A) Shannon index; (B) Observed ASV; (C) Simpson index; (D) Pielou's Evenness index.

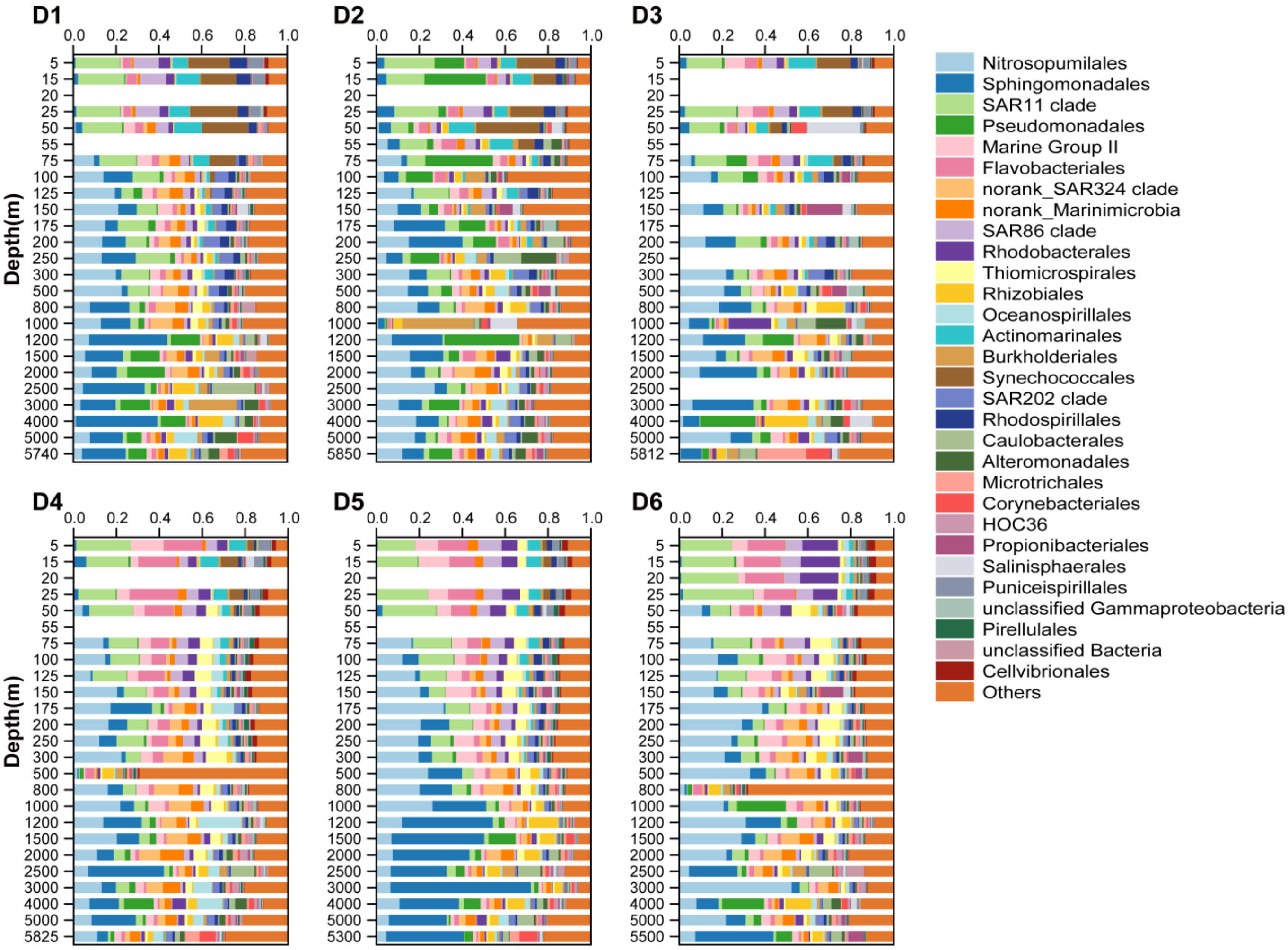

Fig. S2 The microbial community compositions at order level along the D section of the KOE region.

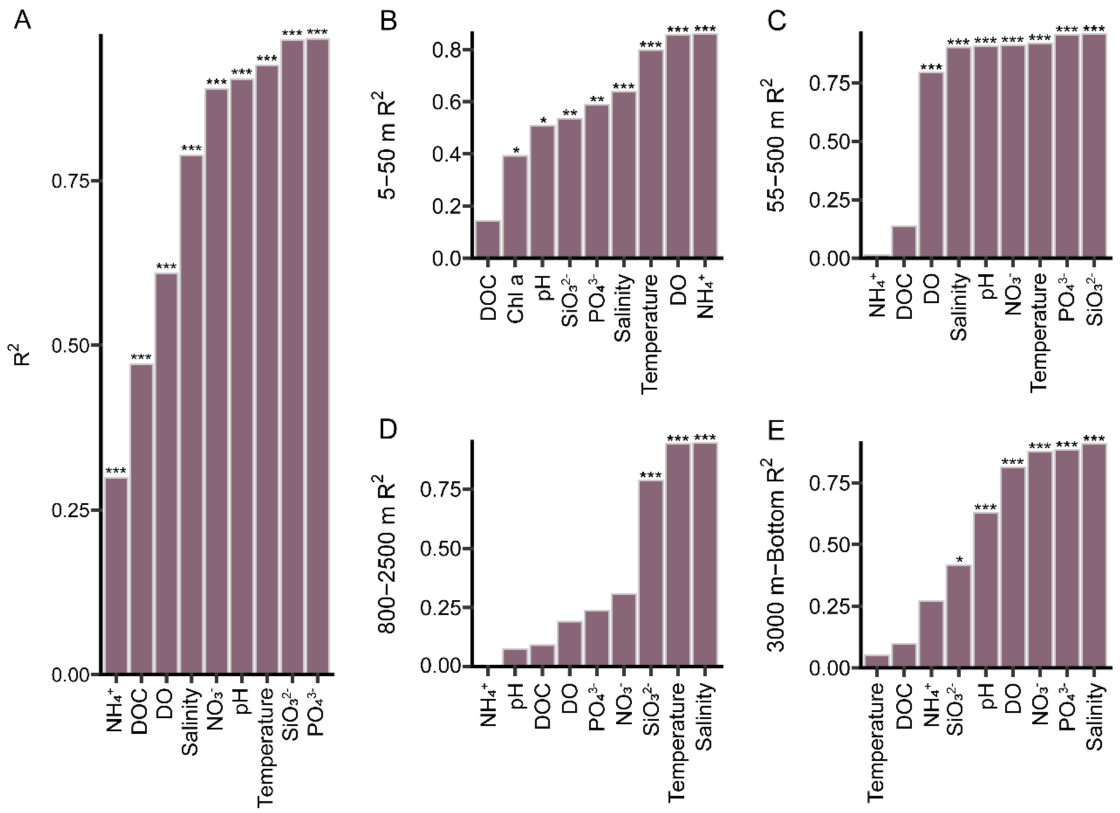

Fig. S3 Explanatory power of environmental factors on prokaryotic community structure across different depth layers. Asterisks indicate the significance levels of correlations based on 999 permutation tests (*$p < 0.05$; **$p < 0.01$; ***$p < 0.001$). (A) all samples across D transect; (B) 5-50 m; (C) 55-500 m; (D) 800-2500 m; (E) 3000 m-bottom.

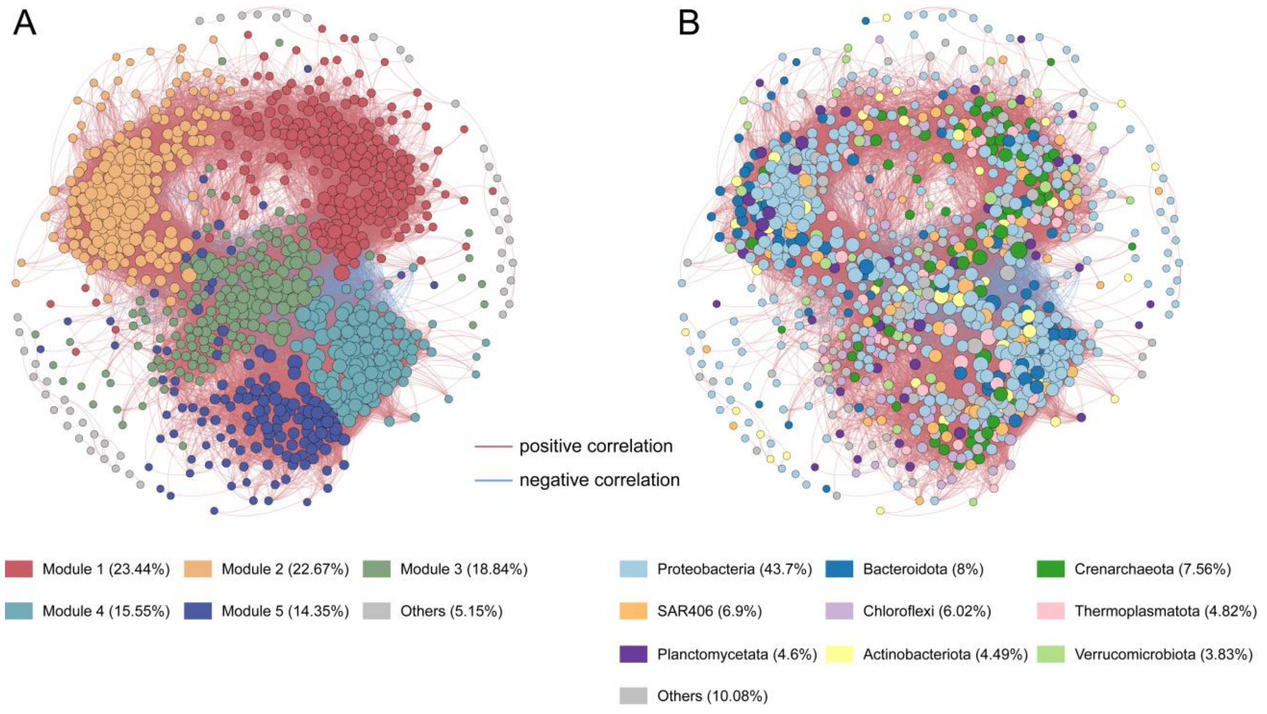

Fig. S4 Co-occurrence Network of microbial community of D transect in KOE region. (A) Coloured by modularity; (B) Colored by taxonomy.

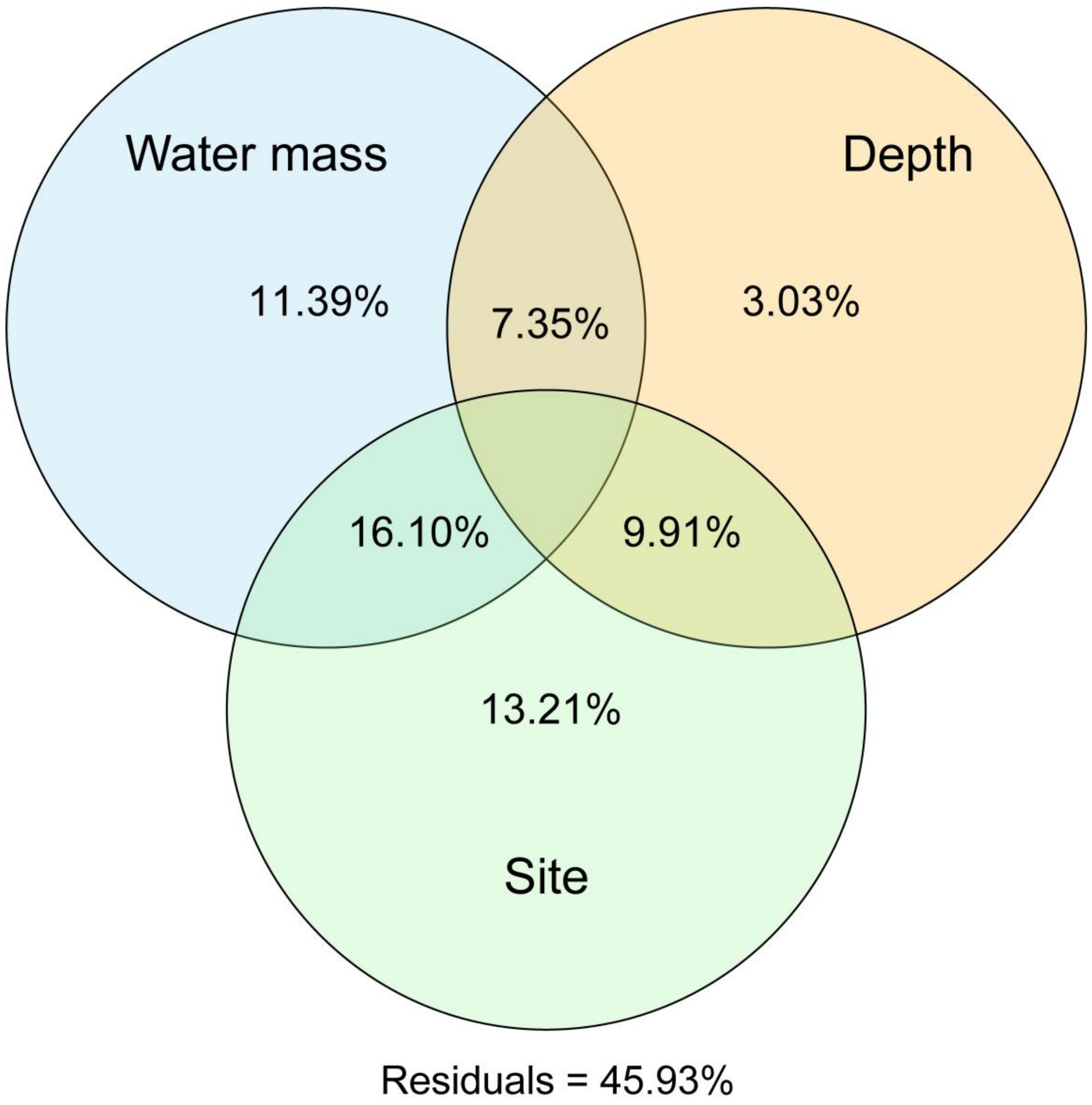

Fig. S5 Venn diagram showing the variance partitioning analysis (VPA) of the explanatory power of depth, horizontal location, and water mass on community structure variation among water masses. Values are adjusted R^2, representing the proportion of total variance in community structure explained by each fraction after correcting for multicollinearity.

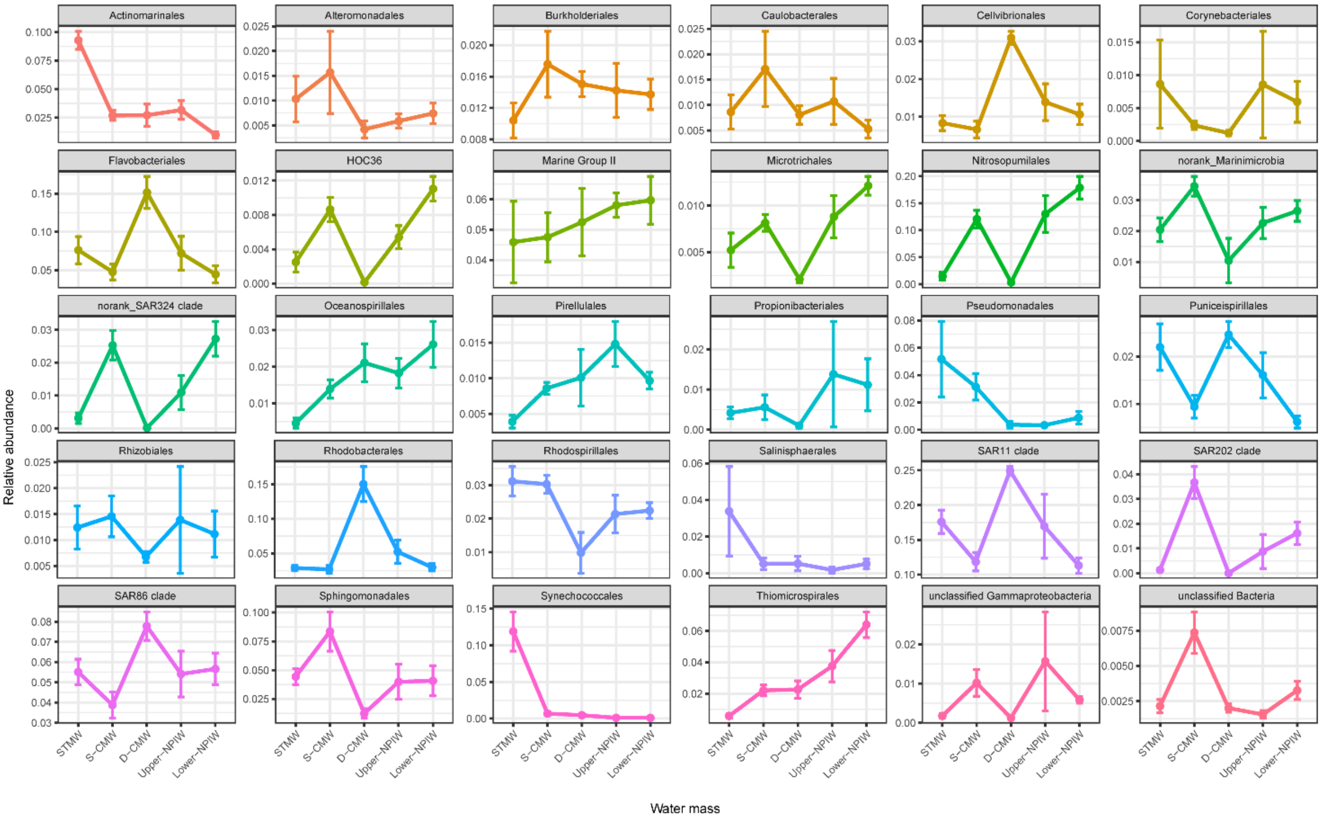

Fig. S6 Relative abundance trends of top 30 abundant orders across five water mass subregions (STMW, S-CMW, D-CMW, Upper-NPIW, Lower-NPIW) along the D transect.

Table S1 Information of co-occurrence networks of microbial community along D transect in KOE region.

Group	Node count	Edge count	Proportion of positive correlations	Average degree	Modularity	Network diameter	Average path length
All layers	913	39745	93.57%	43.532	0.596	9	2.534
D1-D3 5-50 m	270	606	89.93%	4.489	0.903	17	5.746
D4-D6 5-50 m	607	12775	61.86%	42.092	2.027	14	3.495
D1-D3 55-500 m	1935	76942	91.05%	79.527	0.636	11	3.041
D4-D6 55-500 m	1779	104154	76.99%	117.093	0.965	11	2.825
D1-D6 800-2500 m	1259	16148	90.09%	25.652	0.661	15	4.232
D1-D6 3000 m-Bottom	922	7952	93.54%	17.249	0.725	18	4.63

Table S2 Water Mass Distribution.

Water mass		Sample
STMW	STMW	D1-50 m、D2-15 m、D2-25 m、D2-50 m、D2-55 m、D3-25 m、D3-50 m、D3-75 m、D4-5 m、D4-15 m
CMW	S-CMW	D1-100 m、D1-125 m、D1-150 m、D1-175 m、D1-200 m、D1-250 m、D1-300 m、D2-125 m、D2-150 m、D2-175 m、D2-200 m、D2-250 m、D2-300 m、D3-200 m、D3-300 m、D4-50 m、D5-5 m、D5-15 m、D5-25 m
	D-CMW	D5-50 m、D6-5 m、D6-15 m、D6-20 m
NPIW	Upper-NPIW	D3-500 m、D4-75 m、D5-75 m、D5-100 m、D6-25 m
	Lower-NPIW	D1-500 m、D2-500 m、D4-100 m、D4-125 m、D5-125 m、D5-150 m、D5-175 m、D5-200 m、D6-50 m、D6-75 m

Table S3 Information of sampling sites.

Site	Location	Sampling depth	Number of sampling layers
D1	145.2° E，30.4° N	5-5740 m	23
D2	146.2° E，32.4° N	5-5850 m	24
D3	147.6° E，35.0° N	5-5812 m	18
D4	148.8° E，37.0° N	5-5825 m	23
D5	150.0° E，39.0° N	5-5300 m	23
D6	151.2° E，41.0° N	5-5500 m	24
